# Membrane Electrode Assembly Design for High-Efficiency Anion Exchange Membrane Water Electrolysis

**DOI:** 10.34133/research.0907

**Published:** 2025-09-30

**Authors:** Liming Yang, Shengbing Dong, Tao Yang, Jianhe Liu, Shuang Liu, Kang Wang, Enhui Wang, Hongyang Wang, Kuo-Chih Chou, Xinmei Hou

**Affiliations:** ^1^ Institute for Carbon Neutrality, University of Science and Technology Beijing, Beijing 100083, China.; ^2^ State Key Laboratory of Environmental Criteria and Risk Assessment, Chinese Research Academy of Environmental Sciences, Beijing 100012, China.; ^3^ Institute of Steel Sustainable Technology, Liaoning Academy of Materials, Shenyang 110000, China.; ^4^ Beijing Advanced Innovation Center for Materials Genome Engineering, University of Science and Technology Beijing, Beijing 100083, China.

## Abstract

Growing interest in low-cost clean hydrogen production has positioned anion exchange membrane water electrolysis (AEMWE) as a leading sustainable technology. Its appeal lies in compatibility with platinum-group metal-free catalysts, inexpensive anode flow fields, and cost-effective bipolar plates. Recent advances in AEMWE focus critically on optimizing membrane electrode assembly (MEA) design to achieve industrially viable efficiency and durability. Key progress includes component-level innovations, such as developing nonprecious metal catalysts, fabricating anion exchange membranes (AEMs) with high ionic conductivity and alkaline stability, and engineering gas diffusion layers (GDLs) with hierarchical porosity for effective mass transport. Central to improving performance is interfacial engineering within the MEA, which combines catalyst layers (CLs), AEM, and GDLs to reduce ionic/charge transfer resistance and prevent mechanical delamination. A transformative breakthrough involves ordered, gap-free electrode assembly. This approach utilizes strategies such as ionomer-bonded architectures to establish continuous ion-conducting pathways or in situ catalyst deposition directly onto AEM surfaces, creating vertically aligned triple-phase boundaries. These ordered structures maximize catalyst utilization, markedly reduce voltage losses at industrially relevant current densities, and mitigate interfacial degradation during differential-pressure operation. Future advancements require scalable manufacturing of these ordered architectures to bridge material innovations with industrial deployment.

## Introduction

Hydrogen has emerged as an attractive energy carrier for storing and repurposing renewable energy. Currently, 95% of hydrogen production relies on fossil fuel-based steam reforming, which generates substantial CO_2_ emissions (1.3 Gt/year globally). This environmental imperative drives urgent demands for green hydrogen production through water electrolysis powered by renewable sources [1–3]. Low-temperature water electrolysis technologies are typically categorized by electrolyte systems: alkaline water electrolysis (AWE), proton-exchange membrane water electrolysis (PEMWE), and anion exchange membrane water electrolysis (AEMWE) [4,5]. For direct comparison of these technologies’ key characteristics, Table 1 summarizes their operational parameters, material requirements, and techno-economic profiles.

**Table 1.** Comparative analysis of core parameters and performance in three water electrolysis technologies: AWE vs. PEMWE vs. AEMWE

Category	AWE	PEMWE	AEMWE
Schematic diagram	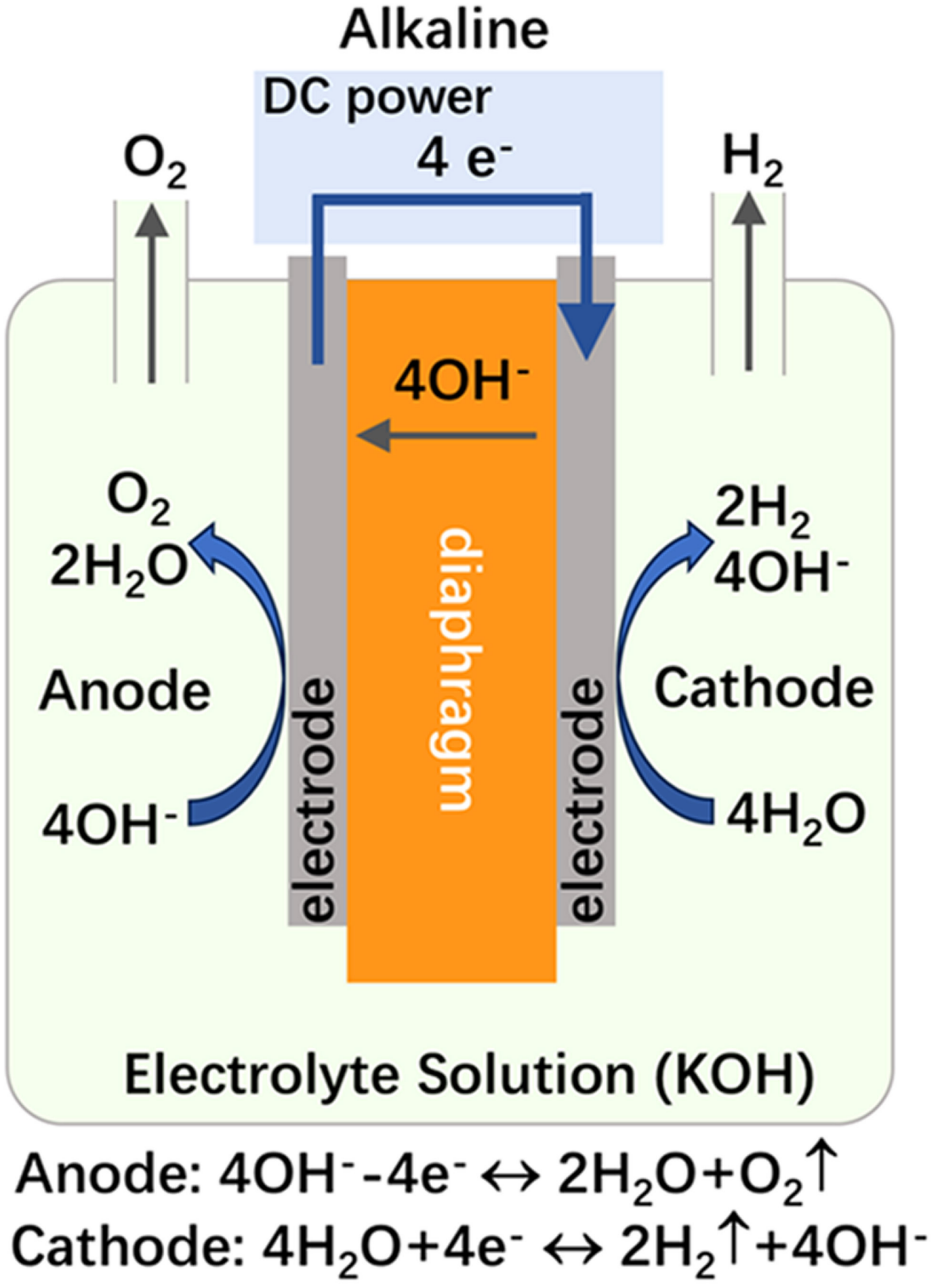	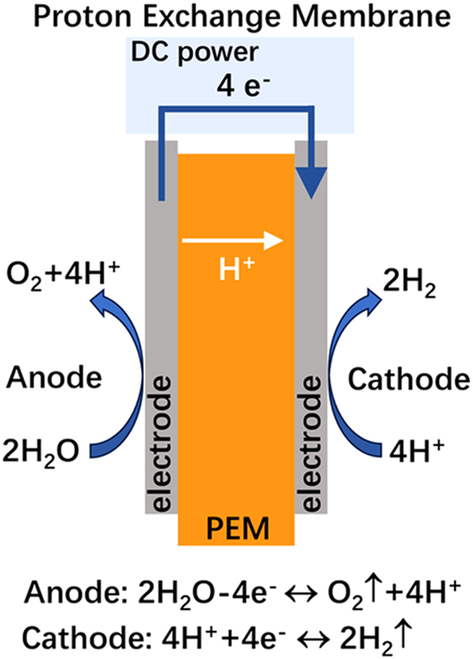	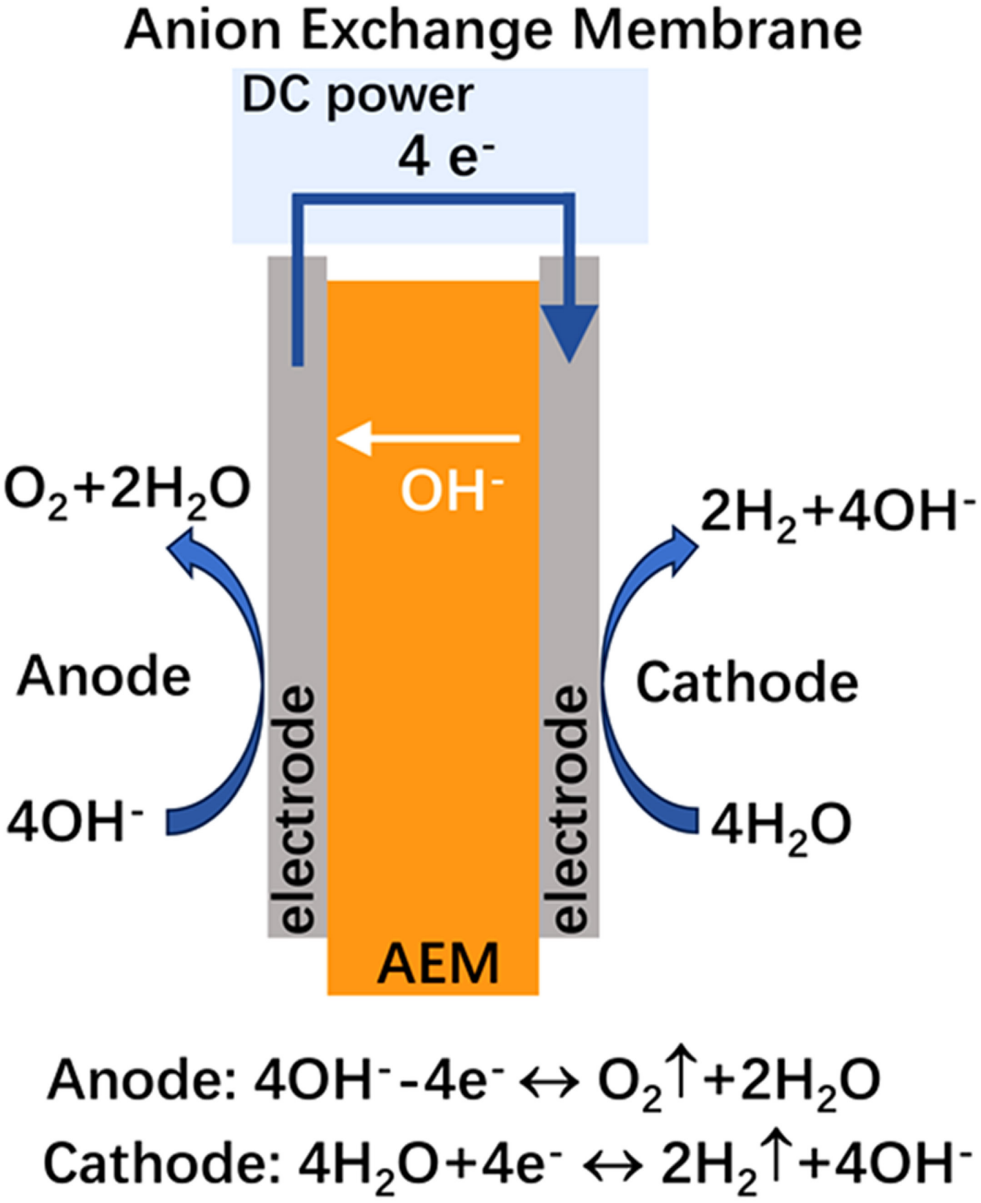
Electrolyte membrane	30% KOH + asbestos membrane	Proton-exchange membrane	Anion-exchange membrane
Gas diffusion layer	Nickel mesh	Titanium mesh/carbon cloth	Nickel foam/carbon cloth
Bipolar plates	Stainless steel/Nickel-coated stainless steel	Platinum/Gold-coated titanium or titanium	Stainless steel/Nickel-coated stainless steel
Operating temperature (°C)	≤90	≤80	≤60
Current density (A/cm^2^)	<0.8	>1	>1
Power consumption (kWh/Nm^3^)	4.5–5.5	4.0–5.0	–
Cell pressure (bar)	<30	<70	<35
Relative volume	1	~1/3	–
H_2_ purity	≥99.5%	≥99.9%	≥99.9%
Service life (h)	60,000	50,000–80,000	>30,000
Single-unit scale (Nm^3^/h)	≤1,000	≤200	–
Technical maturity	Fully industrialized	Initially commercialized	Laboratory stage
Advantages	• Mature technology • No noble-metal catalysts • Low cost • Long-term stability	• Commercialized • High current density • Fast start/stop • High gas purity	• High current density • Low-concentration electrolyte • No noble-metal catalysts • Fast start/stop • High gas purity
Disadvantages	• Limited current density • Pressure difference control required • Strong alkali corrosion • Asbestos hazards	• Noble-metal catalysts • Acidic electrolyte • High component cost	• Limited stability • Under development

AWE systems employ concentrated KOH/NaOH electrolytes (25 to 30 wt%) with nickel-based electrodes separated by asbestos diaphragms. While enjoying mature industrialization and low catalyst costs, their large electrode spacing (2 to 4 mm) fundamentally limits current density (<0.8 A/cm^2^) and causes significant ohmic losses, resulting in bulky systems with high energy consumption (4.5 to 5.5 kWh/Nm^3^). The required gas–liquid separation and alkaline mist management further complicate system integration with intermittent renewables. PEMWE systems address AWE limitations through zero-gap configurations with proton-exchange membrane (PEM), achieving higher current densities (1 to 2 A/cm^2^) and compact designs (1/3 volume of AWE). However, their dependence on platinum-group metal catalysts (IrO_2_ anodes and Pt cathodes) and perfluorinated sulfonic acid membranes (e.g., Nafion) leads to high material costs (>€ 2,000/m^2^ stack). Acidic operation (pH 2 to 4) further accelerates component degradation [6,7]. AEMWE technology synergizes advantages from both systems: it preserves AWE’s noble-metal-free catalysis in alkaline environments while adopting PEMWE’s zero-gap architecture [8–10]. Recent advances demonstrate comparable performance to PEMWE (1.8 to 2.4 A/cm^2^ at 1.8 V) using Fe/Ni-based catalysts and hydrocarbon membranes [11,12]. Nevertheless, the membrane electrode assembly (MEA) is a critical bottleneck. It is the core component of the system, governing ion transport, reaction kinetics, and durability. While substantial progress has been made in developing stable anion exchange membrane (AEM) and efficient catalysts, few reviews systematically address MEA optimization strategies integrating material development, interface engineering, and scalable fabrication.

In this review, the design principles of MEA for high-efficiency AEMWE are systematically deconstructed. The present study commences with an exposition of the foundational architecture and operational mechanisms of MEAs. This is followed by a critical analysis of how innovations at the component level, including catalyst layers (CLs), AEM, gas diffusion layers (GDLs), and their interfacial synergies, collectively dictate device performance. The review synthesizes emerging MEA optimization strategies, with a dedicated focus on overcoming interfacial transport bottlenecks through gap-free ordered electrode architectures. The integration of material science advancements with scalable fabrication paradigms enables the consolidation of the latest breakthroughs in AEMWE technology and the establishment of a framework for bridging laboratory-scale discoveries to industrial deployment. The objective of our analysis is to catalyze interdisciplinary efforts toward next-generation MEA, with the ultimate aim of accelerating the commercialization of alkaline electrolysis as a cornerstone of the green hydrogen economy.

## Fundamentals of MEA in AEMWE

### Composition and working principle

**Fig. 1.**
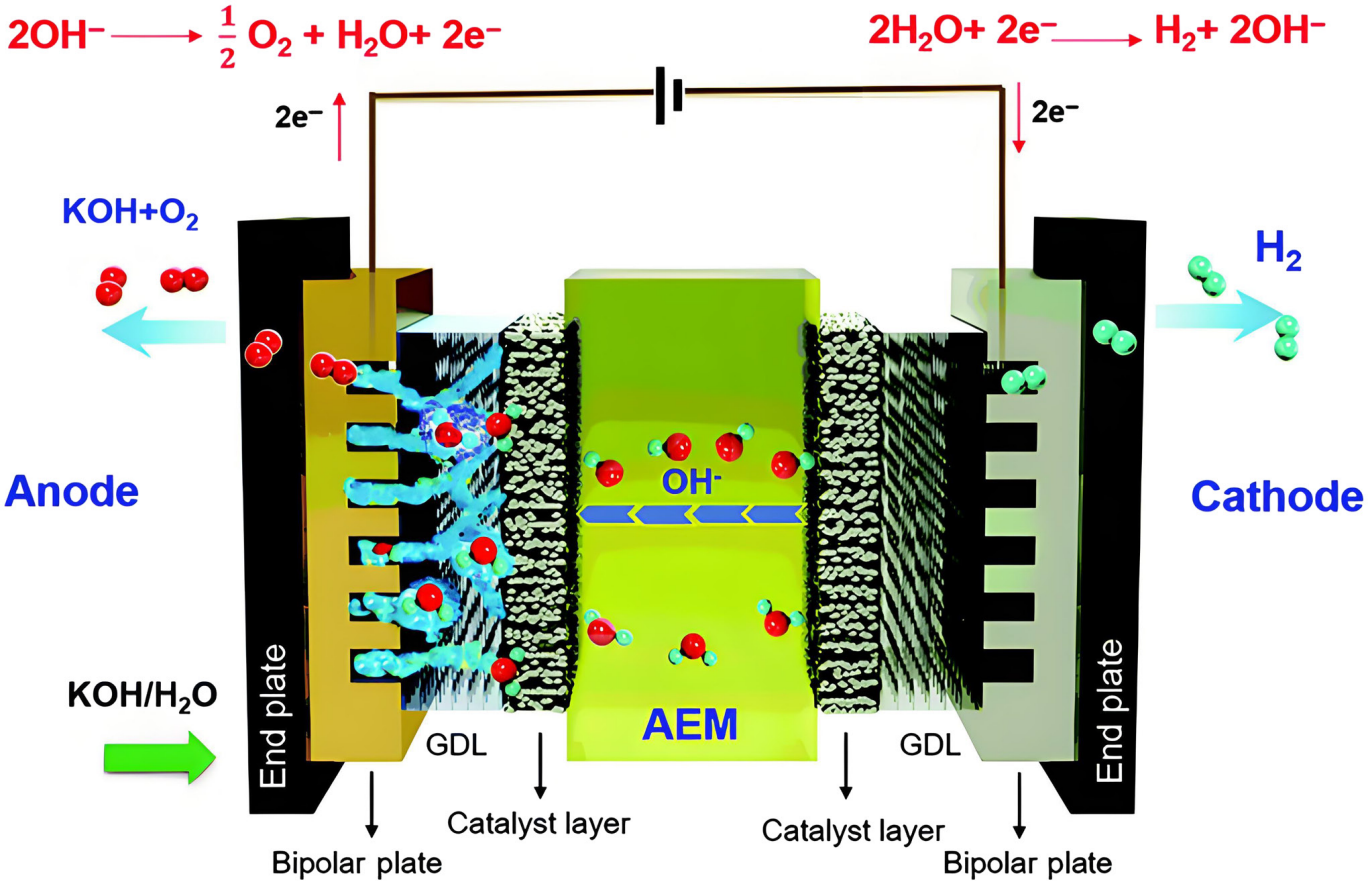
Typical components in AEMWE systems with AEM, GDLs, and BPs [108].

The MEA serves as the electrochemical core of AEMWE, integrating multilayered components that synergistically govern device performance. As illustrated in Fig. 1, the MEA is architecturally composed of a hydroxide-conductive AEM flanked by porous CLs and GDLs, collectively encased between corrosion-resistant bipolar plates (BPs). The AEM, typically a quaternary ammonium-functionalized poly(aryl piperidinium) copolymer, features a dense nonporous microstructure (thickness: 30 to 100 μm) embedded with fixed cationic groups. This design enables selective OH^−^ transport (80 to 150 mS/cm at 60 °C) while blocking gas crossover [H_2_ permeability < 1×10^−14^ mol/(cm·s·Pa)] and electron leakage, thereby permitting zero-gap cell configurations and differential-pressure operation up to 35 bar [13,14]. Adhered to both membrane surfaces via ionomer networks, the CLs (thickness: 10 to 100 μm) establish catalytically active triple-phase boundaries where solid catalysts, liquid electrolytes, and gaseous products interact to drive the hydrogen evolution reaction (HER) and oxygen evolution reaction (OER) at minimized activation overpotentials. Adjacent to the CLs, the GDLs (e.g., microporous Ti felt or Ni foam; porosity > 60%) employ gradient pore structures (macropores: 20 to 50 μm for bulk gas transport; mesopores: 1 to 5 μm for capillary-driven water distribution) to ensure uniform reactant access while preventing electrolyte flooding [15,16]. During operation (Fig. 1), water molecules undergo cathodic reduction (Eq. 1), generating H_2_ gas that diffuses through cathode GDL channels, while OH^−^ ions migrate across the AEM to the anode. There, hydroxide oxidation occurs (Eq. 2), with O_2_ venting via anode GDL pores and electrons circulating externally to complete the energy conversion cycle.

$$\text{Cathode}\;:{\text{2H}}_{2}O+{\text{2e}}^{-}\to H_{2}\;↑+{\text{2OH}}^{-};E{}^{\circ}=0\;V\;\mathrm{vs}.\mathrm{RHE}$$ (1)

$$\text{Anode}\;:{\text{4OH}}^{-}\to O_{2}\;↑+{\text{2H}}_{2}O+{\text{4e}}^{-};E{}^{\circ}=1.23\;V\;\mathrm{vs}.\mathrm{RHE}$$ (2)

$$\text{Total process}\;:{\text{2H}}_{2}O\;\left(l\right)\to {\text{2H}}_{2}\;\left(g\right)+O_{2}\;\left(g\right)$$ (3)

MEA design refers to a systematic optimization process integrating material selection, interfacial engineering, and operational adaptability. It encompasses 3 interrelated dimensions: (a) matching component properties (e.g., catalyst activity, membrane conductivity, and GDL porosity) to basic reaction demands; (b) engineering interfaces (CLs/AEM and CLs/GDLs) to minimize transport resistances (ionic/electronic/mass); and (c) adapting to industrial system requirements (e.g., high current densities and long-term stability in alkaline media).

### Key performance metrics

#### Intrinsic activity

**Fig. 2.**
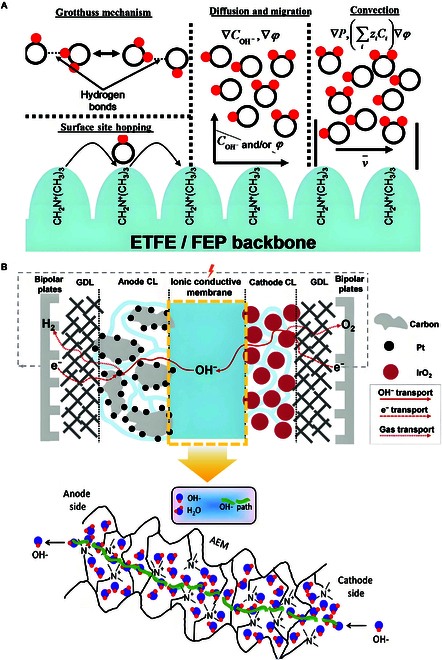
(A) Transport mechanism of OH^−^ in AEM [60]. (B) Transport path diagram of OH^−^, e^−^, and gas in AEMWE systems [5,61].

Both HER and OER are heterogeneous reactions in which electron transfer occurs on the electrode surface. Modification of electrocatalysts is usually aimed at reducing the energy barrier of the reaction, which is observed as a lower overpotential (*η*) and an increase in electrochemical active area (ECSA) in electrocatalysis. Therefore, there are 2 main methods for improving the activity of electrocatalysts: (a) increasing the intrinsic activity of the catalysts [17,18], and (b) increasing the number of active surface sites [19]. There are many methods to measure catalyst activity, and the *η* at a specific current density per electrode area is usually used as the standard to judge (Fig. 2A). These values are difficult to compare because the amount of catalyst supported on the electrode may vary. Another indicator used in some studies is turnover frequency (TOF) (Fig. 2B). TOF refers to the number of catalytic reactions or the number of target products or the number of reactants consumed per unit active site per unit time. For ease of calculation, *JA* is usually used to denote the number of target products or the number of reactants. The equation is:

$$\mathrm{TOF}=\frac{\mathrm{JA}}{\alpha \mathrm{Fn}}$$ (4)

where *J* is the current density at a specific *η*, *A* is the area of the electrode, *F* is the number of electrons in the reaction design, and *n* is the number of moles of active material on the working electrode [20]. However, there are significant inconsistencies in the calculation of TOF, especially in estimating the number of moles of catalyst used [21]. Some authors use the total number of moles of metal atoms in the catalyst, while others use the number of moles of atoms on the surface of the catalyst [22,23]. Therefore, the TOF values reported in the literature do not allow the comparison of catalyst performance between different studies. Besides, it is difficult to obtain accurate TOF values due to the complex composition and surface structure of actual catalysts. How to obtain more accurate TOF values is still a big challenge [24]. In summary, for the purposes of catalyst material research, it seems preferable and simple to consistently report simple current densities at specific *η* values (preferably for the same electrolyte) rather than TOF. In addition, the Tafel equation reflects the kinetic information of the catalytic reaction. The equation is:

$$\eta =a+b\times \text{log}\;\left(j\right)$$ (5)

where *b* is the Tafel slope, *η* is the overpotential, *j* is the current density, and *a* is a constant [25] (Fig. 2C). The Tafel curve describes the rate of current density increasing with the increase of overpotential in steady state, which is one of the important factors to evaluate catalyst activity. The lower the Tafel slope, the less increment of *η* is needed to increase the same current density, which means faster reaction kinetics. The ideal electrocatalyst should have a small Tafel slope at a high current density. Another important parameter is the ECSA of the electrocatalyst [26] (Fig. 2D). The method of increasing ECSA is usually to prepare nano-sized catalysts to achieve maximum exposure of the active surface of the catalysts [27]. However, it is necessary to study in actual AEMWE systems to confirm whether nano-sized catalysts maintain their advantages of high surface area. In the MEA, electrocatalysts need to form conductive networks without hindering the flow of reactants and products.

#### Stability

**Fig. 3.**
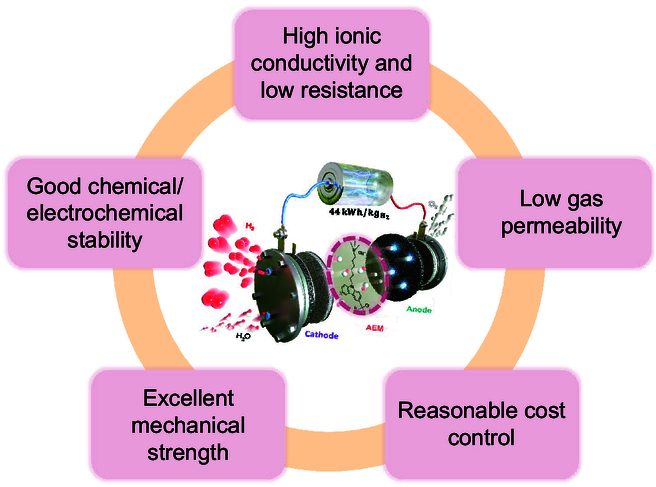
Basic properties of anion exchange membrane [109].

The development of catalysts necessitates simultaneous optimization of electrocatalytic activity and operational stability, particularly under the harsh reducing (HER) and oxidizing (OER) environments inherent to water electrolysis. As illustrated in Table 2, 3 standardized electrochemical methods are widely employed to assess catalyst stability. (a) Chronopotentiometry (CP) [28,29] (Fig. 3A): Voltage fluctuations are monitored at a fixed current density (typically 10 to 100 mA/cm^2^). Smaller voltage drift rates (<2%) indicate superior stability, with industrial-grade catalysts requiring >100 h continuous operation at ≥100 mA/cm^2^. (b) Chronamperometry (CA) [30,31] (Fig. 3B): Current decay is measured under constant applied voltage (e.g., 1.5 to 2.0 V vs. RHE). Acceptable performance degradation thresholds are <5% current loss after over 100 h of testing. (c) Cyclic voltammetry (CV) [32,33] (Fig. 3C): Catalytic durability under dynamic conditions is evaluated through >5,000 CV cycles (e.g., 0.1 to 1.5 V vs. RHE). ECSA retention > 80% post-testing is considered industrially viable. Structural integrity (e.g., phase segregation resistance) and catalytic robustness (e.g., active site retention) must be synergistically maintained, as exemplified by recent advances in nitrogen-doped cobalt oxide (Co_3_O_4_-N) catalysts achieving a current density of 1.0 A/cm^2^ at 1.78 V during 300-h CP testing in 1 M KOH [34].

**Table 2.** Applicable scenarios of testing methods and corresponding industrial standards

Method	Testing conditions	Key metrics	Industrial threshold
CP	10–100 mA/cm^2^, 25–80 °C	Voltage drift rate (mV/h)	<2 mV/h over 100 h
CA	1.5–2.0 V vs. RHE, 0.5–1.0 M KOH	Current retention (%)	>95% after 100 h
CV	5,000 cycles, 0.1–1.5 V vs. RHE	ECSA retention (%)	>80% post-testing

## Component-Level Optimization Strategies

### Catalyst layers

The catalytic efficiency of the HER at the cathode and the OER at the anode is a critical limiting factor for AEMWE systems. Developing nonprecious metal catalysts with low overpotentials (<300 mV@10 mA/cm^2^), high stability (>100 h), and low cost remains a central challenge for advancing the commercial viability of AEMWE systems. Since these systems typically operate in strongly alkaline environments, catalysts must simultaneously exhibit high activity, corrosion resistance, and cost-effectiveness. Contemporary research primarily focuses on replacing noble metals (e.g., Pt and Ir) with transition metal-based alternatives, employing strategies like electronic structure modulation (e.g., Ru single-atom doping [35]), dynamic reconstruction (e.g., phase transitions in Ce-doped composites [36]), and hierarchical structural engineering (e.g., NiFe layered double hydroxide [LDH]/N-doped Co electrodes [37]) to enhance intrinsic activity and stability. Flexible electrocatalysts emerge as a promising solution to these challenges. Their unique structural designs (e.g., 2D ultrathin nanosheets [38,39]) maximize active site exposure and facilitate rapid mass transfer, directly reducing overpotentials in both HER and OER. Coupled with inherent structural stability under prolonged alkaline operation and potential for nonprecious transition metal development, they align with cost-reduction goals, accelerating AEMWE commercialization.

A comparative analysis of catalyst categories (Table 3) further clarifies performance trade-offs under alkaline conditions. Layered bimetallic hydroxides exhibit excellent low-current activity (<300 mV@10 mA/cm^2^) due to adjustable interlayer anions and alkaline resistance, suiting small-scale applications. However, their susceptibility to dissolution limits durability at industrial current densities (>1 A/cm^2^). High-entropy compounds exhibit advantageous properties due to their multielement synergy, which leads to a reduction in metal leaching. Sulfide/phosphide catalysts show promising mass activity but rely on conductive carriers, introducing oxidation risks in OER environments. These distinctions highlight that catalyst selection must align with operational scales: low-cost hydroxides for distributed systems and robust high-entropy alloys for centralized gigawatt facilities.

**Table 3.** Classification and performance summary of AEMWE cathode and anode catalysts

Electrode type	Catalyst category	Typical material examples	Common features	Overpotential
Anode (OER)	Layered bimetallic hydroxides	NiFe-LDH [37,111], CoFe-LDH [112], NiCo-LDH [11], NiFeCo-LDH [113], etc.	• High specific surface area • Interlayer anion adjustable • Strong stability in an alkaline environment • Rich active checkpoints	• NiFe-LDH:250 mV@10 mA**/**cm^2^ [114] • CoFe-LDH: 218 mV@10 mA**/**cm^2^, 276 mV@1,000 mA**/**cm^2^ [112]
	Oxide	LaNiO_3_ [115], NiFe_2_O_4_ [116,117], CoVO [82], Cu*_x_*Co_3-*x*_O_4_ [118], FeNiCoO*_x_*/CoO*_x_* [119], etc.	• Oxygen vacancy abundance • The electronic structure can be modulated by doping • 3D ion transport channel • Alkali corrosion resistance • Suitable for high current density operation	• LaNiO_3_: 361 mV@10 mA**/**cm^2^ [115] • NiFe_2_O_4_: 388 mV@10 mA**/**cm^2^ [116] • FeNiCoO*_x_*/CoO_x_: 221 mV@100 mA**/**cm^2^ [119]
	High-entropy Compound	FeCoNiCuMo [120], NiCoFeCrMn [121], (FeCoNiCrMnCu)_3_O_4_ [122], etc.	• Multielement synergy effect • High configuration entropy enhancement stability • Amorphous/porous structure exposure activity checkpoint	• FeCoNiCuMo: 241 mV@10 mA**/**cm^2^ [120] • NiCoFeCrMn: 317 mV@100 mA**/**cm^2^ [121] • (FeCoNiCrMnCu)_3_O_4_: 241.4 mV@100 mA**/**cm^2^ [122]
Cathode (HER)	Transition metal alloy	NiMo [123,124], Ni_4_Mo [125], NiCo [126], NiAl [10], Ru–Co_2_Ni [127], etc.	• Low hydrogen adsorption energy • High conductivity • Suitable for alkaline media	• NiMo: 85 mV@10 mA/cm^2^ [123] • NiCo: 140 mV@10 mA/cm^2^ [126] • NiAl: 66 mV@10 mA/cm^2^, 213 mV@100 mA/cm^2^ [10] • Ru–Co_2_Ni: 35 mV@10 mA/cm^2^ [127]
	Sulfide/Phosphide	Mo-NiS_2_ [128], Co-1T-MoS_2_ [129], F-CoP [130], Co_9_S_8_@CoMoP*_x_* [131], etc.	• Marginal activity checkpoint exposure • Strong corrosion resistance • It needs to be combined with the carrier to improve stability	• Mo-NiS_2_: 306 mV@100 mA/cm^2^ [128] • Co-1T-MoS_2_: 118 mV@10 mA/cm^2^, 239 mV@200 mA/cm^2^ [129] • F-CoP: 90 mV@1,000 mA/cm^2^ [130] • Co_9_S_8_@CoMoP: 226 mV@500 mA/cm^2^ [131]
	Carbon matrix composite	Pt@Co-NPC [132], Fe_2_P-Co_2_P/NPC [133], Ru/Ni-N_4_C-300 [66], Li_3.0_RuSn_0.8_ NWs [79], etc.	• High electron mobility • Multistage pore structure and high specific surface area • Heteroatom doping and electronic structure regulation	• Pt@Co-NPC: 34 mV@10 mA/cm^2^ [132] • Fe_2_P-Co_2_P/NPC: 175 mV@1,000 mA/cm^2^ [133] • Ru/Ni-N_4_C-300: 15 mV@10 mA/cm^2^ [134] • Li_3.0_RuSn_0.8_ NWs: 66 mV@100 mA/cm^2^ [79]
	High-entropy compound	High-entropy oxychalcogenide (HEOC) [135], Pt/(FeCoNiCrAl)_3_O_4_ [136], etc.	• Multiactivity checkpoint coordination • Electronic structure optimization • Inhibition of metal dissolution in alkaline media	• High-entropy oxychalcogenide (HEOC): 57 mV@10 mA/cm^2^ [135] • Pt/(FeCoNiCrAl)_3_O_4_: 22 mV@10 mA/cm^2^ [136]

Notably, most lab-developed catalysts only maintain low current densities (<500 mA/cm^2^) for short durations (<500 h) under mild conditions (1 M KOH, 25 °C) [40,41], primarily due to in situ degradation. Key internal degradation factors include morphology changes [42], surface poisoning [43,44], and metal dissolution [45]. In HER, activity declines may stem from decomposition [46,47], pinch spalling [48], hydroxide formation [49], nanoparticle aggregation, or redeposition of dissolved cations [50]. In OER, catalysts often act as “pre-catalysts”, transforming into metal (oxy)hydroxides as the true active phase [51,52]. However, this reconstruction can accelerate dissolution, undermining stability. In a word, in order to improve the stability of electrocatalysts in HER and OER processes, researchers need to deeply understand their degradation mechanisms and take effective measures to improve the performance and life of catalysts. This may include the reasonable design of catalysts, the optimization of preparation methods, and the adjustment of reaction conditions.

### Anion exchange membrane

AEM is the crucial core component of AEMWE systems for hydrogen production. Its efficient operation and long-term stability are essential prerequisites for the commercial viability of AEMWE’s systems. The AEM performance metrics in Table 4 reveal a clear conductivity–stability–cost trilemma. German DURAION leads in ionic conductivity and longevity, but its high production cost restricts adoption to high-performance niches. In contrast, China’s W-25 membrane balances conductivity and affordability, making it a pragmatic choice for mid-scale electrolyzers. For industrial deployment, systems prioritizing continuous operation (>5,000 h) should prioritize DURAION or Aemion+, while cost-sensitive applications may opt for W-25 or Hydrergy membranes despite slightly lower conductivity.

**Table 4.** Market overview of AEM for electrolysis

Membrane name	Company name	Country	Performance parameters
W-25	Huizhou EVE Hydrogen Energy Co., Ltd.	China	• Thickness 25 ± 2 μm • Ionic conductivity ≥ 150 mS/cm (80 °C, 100% relative humidity [RH]) • Tensile strength ≥ 30 MPa • Resistant to 1 M NaOH solution for 5,000 h
W-75	Huizhou EVE Hydrogen Energy Co., Ltd.	China	• Thickness 75 ± 5 μm • Ionic conductivity ≥ 130 mS/cm (80 °C, 100% RH) • Resistant to 1 M NaOH solution for 5,000 h
Alkymer	Huizhou EVE Hydrogen Energy Co., Ltd.	China	• Polyaromatic backbone + piperidine cation structure, ionic conductivity ≥ 150 mS/cm • Annual production capacity 5,000 m^2^
Carbon hydrogen bond resin membrane	Jiangsu Cleanergy New Energy Technology Co., Ltd.	China	• Thickness 50 μm • Tensile strength > 65 MPa • Adaptable to fluctuating power scenarios
DURAION	Evonik (DURAION)	Germany	• Ionic conductivity > 200 mS/cm • Service life > 12,000 h in 1 M KOH solution
Aemion+	Ionomr Innovations	Canada	• Ultra-stable AEM • Resistant to strong acid and alkali environments • Extended lifespan • Compatible with noble metal-free systems
FAAM	Fumatech BWT GmbH	Germany	• Higher heating value efficiency 85% at 0.8 A/cm^2^ • Low swelling rate • Superior mechanical strength
Sustainion X37	Dioxide Materials	USA	• Thickness 50 μm • Low sheet resistance • Brittle under dry conditions
PiperION	Versogen	USA	• Ionic/chemical stability • Limited mechanical strength
A201	Tokuyama	Japan	• Carbon-hydrogen backbone + quaternary ammonium groups • Dense ion channel network
Enapter AEM	Enapter	Germany	• Current density > 1 A/cm^2^@1.8 V • Adapt to high-efficiency electrolyzers
Hydrergy	Carbon Harmony Tech	China	• Ionic conductivity ~160 mS/cm • Swelling rate 5% • Mechanical strength > 40 MPa (80 μm)
Novamea series	Novamea	Switzerland	• Integrated AEM electrolysis/fuel cell/CO_2_ reduction technology • Systematic electrode optimization

These membranes are composed of 2 critical components: (a) a covalently cross-linked polymer backbone that provides structural integrity and dimensional stability [53,54] and (b) functional cationic groups (e.g., quaternary ammonium [55,56], imidazole [57,58], or quaternary phosphine [51,59], etc.) that facilitate hydroxide ion (OH^−^) transport through electrostatic attraction and hydrophilic channel formation. Regarding the transport mechanism of OH^−^ in AEMs, it is currently widely believed to be similar to the proton conduction mechanism in PEMs, primarily involving the Grotthuss mechanism, diffusion and migration mechanism, and convection mechanism [60] (Fig. 2A). The Grotthuss mechanism involves the formation of hydrated OH^−^ ion clusters through hydrogen bonding between OH^−^ ions and water molecules in the solution. Upon coming into proximity with surrounding water molecules, these clusters undergo a process of restructuring, characterized by the formation of new hydrated OH^−^ ion clusters with other water molecules. This process involves the breaking and rearranging of hydrogen bonds, resulting in the creation of novel hydrated ion clusters. The movement of OH^−^ ions within the electrolyte is driven by the continuous formation and dissolution of hydrogen bonds. The diffusion and migration mechanism is characterized by the movement of OH^−^ ions in an aqueous solution, driven by concentration and potential gradients. The convection mechanism is initiated by the binding of OH^−^ ions to water molecules during transport, resulting in the directed movement of water molecules and the generation of a water pressure gradient. This process, in turn, leads to convection. In actual operating conditions, OH^−^ transport behavior is the result of the combined effects of multiple transport mechanisms (Fig. 2B). The OH^−^ conduction mechanism is contingent upon the synergistic action of cationic headgroups and hydrophilic pathways. The presence of positively charged functional groups has been shown to generate an electric field, a phenomenon that has been observed to attract OH^−^ ions while repelling cations within the electrolyte. The direction of OH^−^ migration is facilitated by ion-exchange sites, which act as conduits through optimally designed transport channels [61].

In the industrial application of hydrogen production by AEMWE systems, AEM needs to meet the following requirements (Table 5): (a) high ionic conductivity and low resistance to ensure the transfer of OH^−^, reactant, and carrier fluid between anode and cathode [62,63]; (b) good chemical/electrochemical stability, keeping long-term stability in pure water/low concentration alkaline aqueous solution when the hydrogen production system runs; (c) low gas permeability, preventing direct contact between cathode and anode gases, ensuring high gas purity and enabling higher gas production pressures; (d) excellent mechanical strength and dimensional stability, keeping good support, tensile resistance, and swelling rate during operation [64]; and (e) reasonable cost control, which is convenient for industrial production (Fig. 3). Unfortunately, to some extent, there is a compromise between their mechanical strength and ionic conductivity. Although increasing the loading of functional groups may be beneficial to the conductivity of hydroxides, it increases the absorption of water and thus reduces the mechanical stability. On the contrary, the lower loading of functional groups may lead to the lower ionic conductivity, which may lead to the degradation of performance. Therefore, meeting the above requirements at the same time is challenging, and the trade-off of the basic properties of the membrane must be carefully designed [5].

**Table 5.** Key performance requirements for AEMs in industrial AEMWE applications

Key performance parameter	Industrial requirement	Test conditions
Ionic conductivity	>120 mS/cm at 60 °C	Liquid water environment, 100% relative humidity
Chemical stability	>5,000 h in 1–2 M KOH at 60–80 °C	Long-term immersion in alkaline solutions
Gas barrier properties	H_2_/O_2_ crossover rate < 1%	Gas permeation under 30 bar pressure differential
Mechanical robustness	Tensile strength > 30 MPa, Young’s modulus > 500 MPa	Tensile testing at 23 ± 2 °C, 50% ± 5% RH
Cost control	<$50/m^2^ for large-scale production	Annual output of 100,000 m^2^

### Gas diffusion layers

GDLs are critical components in AEMWE systems, facilitating efficient electron conduction, reactant/product transport, and mechanical support under high-current-density operation. To meet industrial requirements, GDLs must balance high electrical conductivity (interfacial contact resistance [ICR] < 10 mΩ·cm^2^), hierarchical porosity (bulk porosity > 60% with micropores < 10 μm), and robust mechanical stability (tensile strength > 30 MPa), while resisting degradation in alkaline environments (e.g., 1 to 2 M KOH).

As shown in Table 6, nickel foam (NF) has emerged as the mainstream material for GDLs due to its cost-effectiveness and superior conductivity. However, surface roughness optimization via laser etching or plasma spraying is required to reduce ICR. For high-current-density scenarios (>1 A/cm^2^), carbon fiber-based materials (e.g., Sigracet carbon paper) are preferred owing to their lightweight and high conductivity, but they are more expensive and face challenges in terms of oxidation resistance. Recent studies have improved the adaptability of GDLs through gradient pore structure designs (large pores toward flow fields and small pores toward CLs) and dynamic-responsive materials (e.g., shape-memory alloys). These improvements enable AEMWE systems to maintain a low voltage decay of less than 50 μV/h under industrial-grade current densities [65,66]. In addition, Lei et al. [67] proposed in their review that an ideal GDL should have a layered pore structure to balance hydrodynamics and charge transport. This design strategy has been shown to improve the utilization of the catalytic layer and reduce the ohmic resistance. The future development direction will center on the large-scale preparation of composite-based GDLs (e.g., carbon nanotube/graphene composite carriers) and the optimization of compatibility with novel ionomers. This will improve operational adaptability and service life, and promote the commercialization of AEMWE technology.

**Table 6.** Key characteristics and performance metrics of GDLs for AEMWE systems

Category	Exemplary materials	Structural features	Application scenario
Metal-based	Nickel foam, stainless steel felt, nickel wire mesh	• High electrical conductivity (>100 S/cm), high porosity (70%–90%) • Resistance to alkali corrosion • Suitable for anode side (OER)	Industrial-scale AEMWE stacks
Carbon-based	Carbon fiber paper (e.g., Sigracet); graphene composite	• Lightweight, low density (0.4–0.8 g/cm^3^) • Suitable for the cathode side (HER)	Lab-scale or low-power systems
Composite	Carbon-nickel composites; carbon nanotube/graphene composite carriers; metal fiber sintered layer	• Layer pore design (microporous layer pore size < 10 μm, base layer pore size 50–100 μm) • Balance mass transfer and conductivity	High performance or extreme operating conditions

### MEA interface

**Fig. 4.**
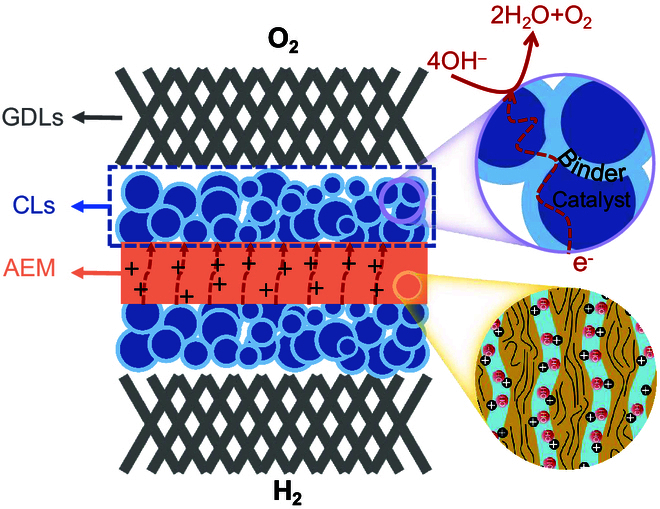
Schematic diagram of the interface of GDLs/CLs/AEM in MEA.

The MEA in AEMWE systems hinges critically on the rational design of CLs, AEM, GDLs, and their interfacial interactions (Fig. 4). These interfaces, encompassing CLs/AEM and CLs/GDLs junctions, govern mechanical integrity, charge transfer (e.g., electrons and OH^−^), mass transport (e.g., water and gas diffusion), and thermal management, directly dictating overall efficiency and durability [68,69]. Traditional MEA fabrication involves spraying catalyst slurries onto GDLs, followed by hot-pressing to bond components. However, poor interfacial adhesion, particularly at the CLs/AEM interface, can disrupt charge transfer pathways, starve active sites of OH^−^, and degrade catalyst utilization by up to 40% due to delamination or void formation [70,71]. A key challenge lies in minimizing ICR. This requires optimizing AEM polymer formulations, such as tailoring the ionomer content to 10 to 30 wt.% to balance ion conductivity and mechanical strength. It also requires developing compatible electrode binders or binder-free strategies, such as growing catalysts directly on AEM surfaces. Equally critical is the CLs/GDL interface, which dominates mass transfer resistance. GDLs must rapidly expel generated gases (H_2_/O_2_) and manage water flux to prevent electrode flooding or drying. Suboptimal pore structure or compression-induced GDL surface roughness creates microgaps at interfaces, elevating ohmic losses [72]. Therefore, the design of the GDLs/CLs interface structure in the membrane electrode is also important to enhance the overall performance of MEA.

## MEA Design Strategies

**Fig. 5.**
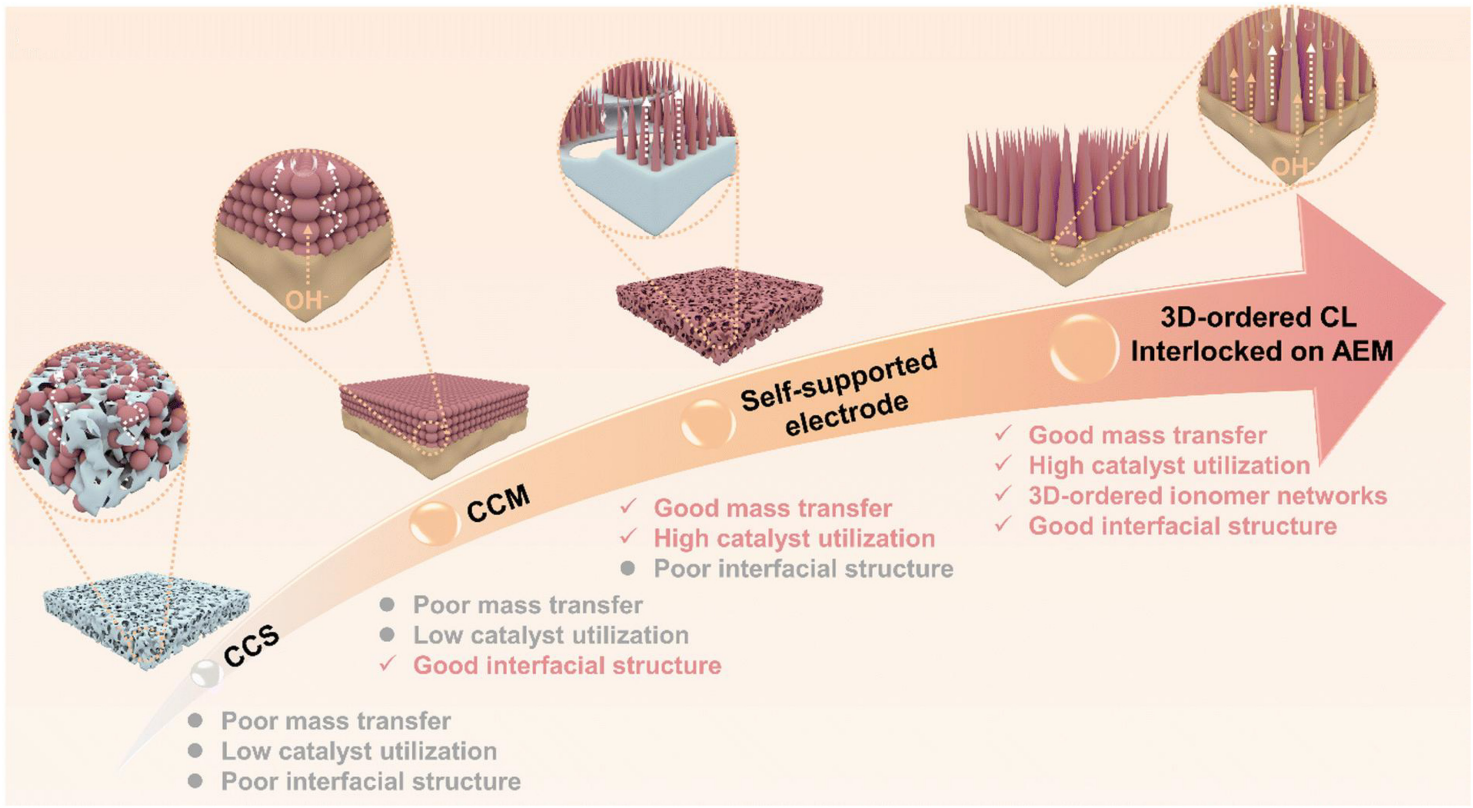
Comparison of advantages and disadvantages of CLs prepared by different methods: schematic illustration of performance differences between conventional CCS, CCM, self-supported electrode, and 3D-ordered CL interlocked on AEM [106].

The design and fabrication of MEA are pivotal in determining the efficiency, durability, and scalability of AEMWE systems. The range of current MEA preparation methods encompasses conventional techniques, including catalyst-coated substrates (CCSs) and catalyst-coated membranes (CCMs), as well as ionomer-free self-supporting catalyst integration and advanced ordered membrane-electrode architectures (Fig. 5 and Table 7). Each approach seeks to optimize interfacial contact, ionic/electronic conductivity, and mass transport while addressing challenges such as catalyst delamination, membrane swelling, and manufacturing scalability.

**Table 7.** Comparison of MEA design strategies for AEMWE systems

MEA design strategy		Core design objectives
1. Coating methods	CCS	• Scalable fabrication • Low-cost mass production
	CCM	• Tighter CLs/AEM interfacial contact • Reduced catalyst loading
2. Ionomer-free self-supporting electrodes	–	• Eliminate binder-induced transport barriers • Enhance electron/mass transfer
3. Ordered membrane electrodes	Nanoscale imprinting	• Optimize ion/ mass transport via ordered channels • Maximize active site exposure
	Integrated membrane electrodes	• Seamless CLs/AEM interface • Minimize interfacial resistance
	3D interlocked interfaces	• Gap-free CLs/AEM contact • Vertical alignment of transport channels

### Coating method

**Fig. 6.**
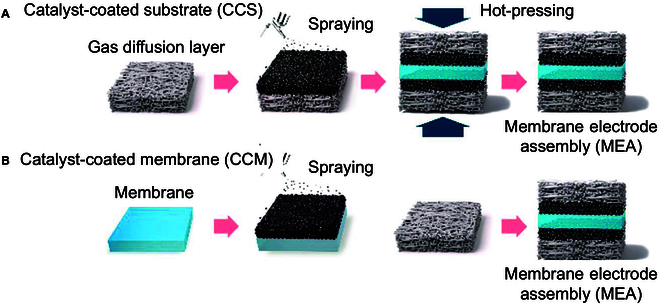
Process flow diagram for (A) CCS and (B) CCM [110].

Coating methods (CCS/CCM) focus on scalable fabrication of basic MEA architectures, balancing catalyst utilization and interfacial contact. The traditional coating preparation process for AEM membrane electrodes is mainly divided into 2 types, namely, CCS and CCM methods, and the main difference between the two is the loading object of the CL [73,74] (Fig. 6).

#### Catalyst-coated substrate

**Fig. 7.**
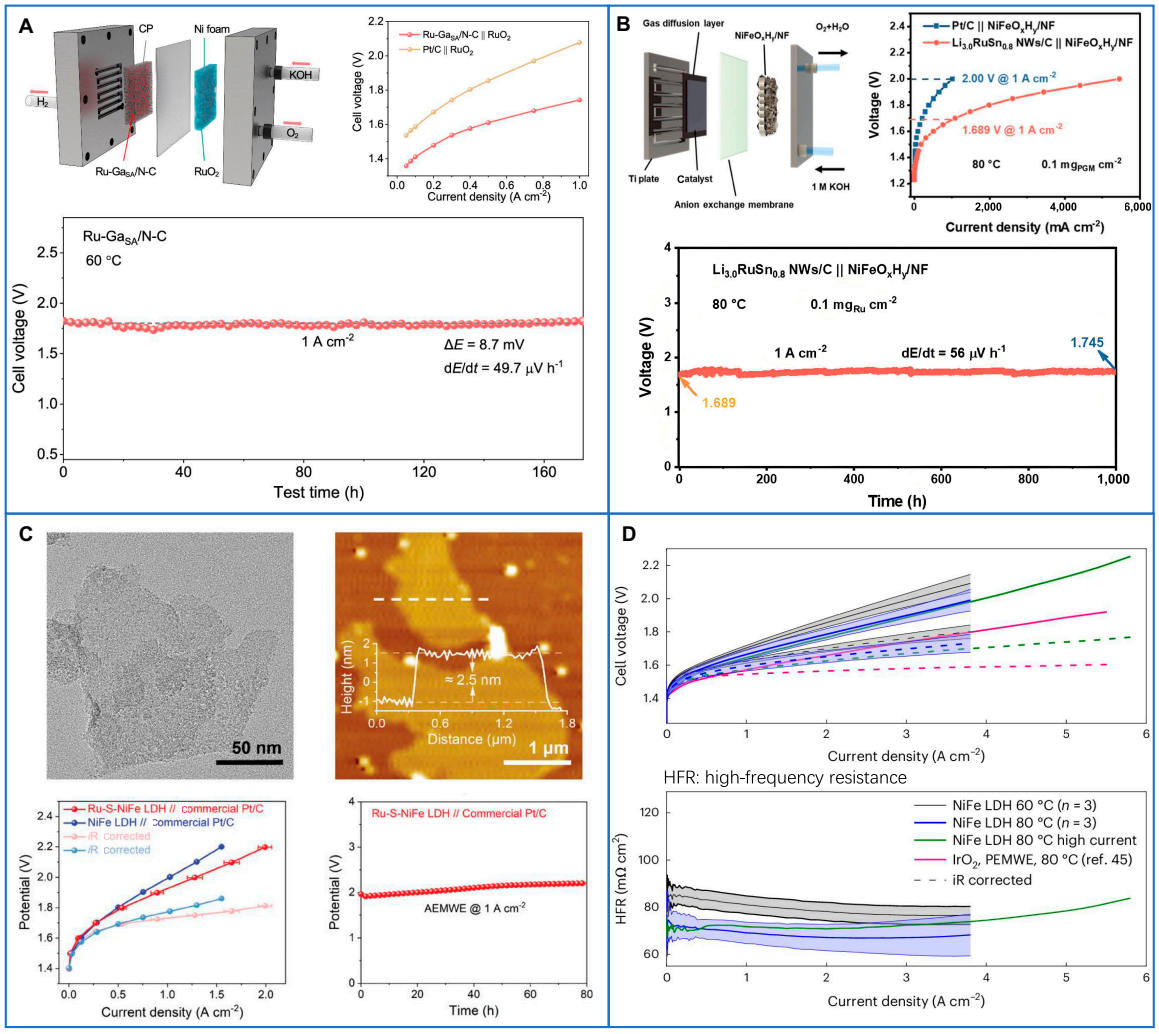
Preparation of MEA of AEMWE by the CCS method. AEMWE schematic, polarization curve, and chronopotentiometric measurement curve of (A) Ru-GaSA/N-C [78] and (B) Li_3.0_RuSn_0.8_NWs/C [79]. Preparation of MEA of AEMWE by the CCS method. (C) TEM morphology and thickness, AEMWE polarization curve, and stability test of Ru-S-NiFe LDH [35]. (D) AEMWE single-cell polarization curve measurement and corresponding high-frequency resistance curve of NiFe LDH catalyst [11].

The CCS method involves depositing catalyst slurries onto GDLs via screen printing, blade coating [75], or spray deposition [76] to form gas diffusion electrodes, which are subsequently hot-pressed with the AEM at 50 to 80 °C under 2 to 5 bar pressure (Fig. 6A). Although CCS is operationally straightforward and compatible with roll-to-roll manufacturing, there are still challenges, such as catalyst infiltration into GDL pores, which leads to 15% to 30% blockage, and interfacial delamination under mechanical stress. These issues result in reduced active site accessibility and elevated ICR [77]. Researchers have focused on addressing these limitations through catalyst design and interfacial engineering. For example, Zhou et al. [78] synthesized a Ru-GaSA/N-C catalyst using a 2-step process. In this process, electron-deficient and oxyphilic Ga single atoms served as electronic bridges that synergized with Ru clusters. This induced strong metal-support interactions and enhanced the catalyst’s ability to water splitting. The fabrication of the AEMWE device involved the application of Ru-GaSA/N-C onto carbon paper for the cathode and RuO_2_ onto NF for the anode, followed by their integration with a commercial AEM (X37-50). It demonstrated stability at a high current density of 1 A/cm^2^ and maintained a degradation rate of only 49.7 μV/h over 170 h (Fig. 7A). In another study, Mao et al. [79] developed an electrochemical lithiation strategy to insert Li into the lattice of sub-2-nm Ru-Sn nanowires, with partial dissolution during electrochemical operation to improve alkaline HER activity. The Li_3.0_RuSn_0.8_ NWs/C catalyst, in conjunction with a NiFeO*_x_*H*_y_* anode supported on NF substrates, facilitated the development of an industrial-scale AEMWE single cell. The device demonstrated 1 A/cm^2^ at an ultralow cell voltage of 1.689 V in 1.0 M KOH at 80 °C. Moreover, this configuration exhibited outstanding stability, operating continuously for 1,000 h at 1 A/cm^2^ with a voltage decay of merely 56 μV/h (Fig. 7B). These advancements demonstrate how tailored catalyst architectures and optimized deposition techniques can mitigate traditional CCS drawbacks, balancing scalability with performance in next-generation electrolyzers.

#### Catalyst-coated membrane

The CCM method directly deposits catalysts onto AEM surfaces via transfer printing [53] or ultrasonic spraying [80], bypassing the need for post-assembly hot-pressing (Fig. 6B). This strategy ensures intimate CLs/AEM contact, achieving ICR values below 10 mΩ·cm^2^ and reducing catalyst loading by 30% to 50% compared to CCS. Innovations such as solvent-resistant hydrocarbon membranes functionalized with cross-linked poly(aryl piperidinium) networks exhibit >95% dimensional stability during spraying, addressing historical challenges of membrane swelling [81]. High-throughput techniques like flexographic printing enable the deposition of ultrathin CLs (5 to 10 μm) with 90% catalyst utilization. However, prolonged solvent exposure during CCM fabrication risks delamination at current densities exceeding 3 A/cm^2^, necessitating advanced interfacial bonding strategies. For instance, Zhu et al. [35] synthesized a Ru-S-NiFe LDH catalyst via a one-pot hydrothermal reaction, simultaneously doping Ru single atoms and S anions into NiFe LDH. The Ru atoms enhanced electrical conductivity, while S anions accelerated electrochemical reconstruction and mitigated Ru overoxidation. Spray Pt-C and Ru-S-NiFe LDH onto the AEM as the cathode and anode catalysts, respectively, then assemble with blank nickel felt as the GDLs. The resulting AEMWE single cell exhibited high stability for over 80 h at 1.0 A/cm^2^ (Fig. 7C). In another study, Klingenhof et al. [11] developed NiX (X = Fe, Co, and Mn) LDH catalysts integrated into CCM, markedly boosting AEMWE performance. The optimized system achieved current densities comparable to PEM electrolyzers at 60 and 80 °C, along with stability exceeding 110 h (Fig. 7D). These advancements demonstrate the potential of customized catalyst architectures to improve the efficiency and durability of CCM-based systems. However, challenges related to scaling precision deposition and mitigating solvent-induced degradation remain critical for industrial adoption.

In summary, the technical principles, advantages, and limitations of CCS and CCM are comprehensively compared in Table 8. While CCS demonstrates compatibility with roll-to-roll manufacturing and simplified operational workflows, it faces persistent challenges such as catalyst infiltration into GDL pores (>20% loss) and interfacial delamination under high current densities (>2 A/cm^2^). In contrast, CCM markedly reduces catalyst waste (<10% loss) and achieves tighter interfacial contact (ICR < 10 mΩ·cm^2^) by directly depositing catalysts onto the membrane surface. While CCS prioritizes gigawatt-scale production with moderate efficiency, CCM offers higher performance (e.g., 50% lower catalyst loading) for compact applications like distributed renewable integration. Neither method fully resolves the balance between mass transport efficiency and long-term durability, underscoring the need for scenario-specific optimization in industrial AEMWE deployments.

**Table 8.** Comparison of CCS and CCM performance, advantages, and disadvantages

Contrast dimension	CCS	CCM
Technical principle	The catalyst slurry is coated on the surface of GDLs and combined with AEM by hot pressing	The catalyst is directly coated on both sides of the AEM and then combined with the GDLs by hot pressing
Catalyst utilization rate	Lower (about 20%–50%)	Higher (up to 85%–95%)
ICR	Higher (>15 mΩ·cm^2^)	Lower (<10 mΩ·cm^2^)
Advantage	• Simple process, compatible with roll-to-roll production • AEM is not easy to deform	• Low interface resistance and stable performance • Lower catalyst loading (0.4–1 mg/cm^2^) • Suitable for high current density (>3 A/cm^2^)
Disadvantage	• Serious waste of catalysts • Hot pressing is prone to AEM micro-cracks • The interface is easy to peel off after long-term operation	• High risk of AEM swelling (requires solvent-tolerant formulation) • The process window is narrow (the water–alcohol ratio is strictly controlled) • High equipment costs

### Ionomer-free self-supporting electrodes

Ionomer-free self-supporting electrodes aim to eliminate binder-induced transport barriers, optimizing electron conduction and mass transfer at the CLs/GDLs interface. Conventional MEA preparation techniques typically spray inks formulated with catalyst powders and binders onto GDLs or AEM. In the last decade, a new type of self-supported catalytic electrode has been widely investigated. As the name suggests, the catalytically active material is directly grown in situ on a conductive GDL substrate. Compared with conventional powder catalytic electrodes, self-supported catalytic electrodes have the following advantages: (a) The ability to effectively avoid the use of binders, thus effectively enhancing the mass transfer process. Substantially reduce the electrolyte/electrode resistance, while being able to expose more active sites [82]. (b) Realize the close combination of catalyst and conductive substrate, which ensures the electron transfer efficiency between active material and substrate. Effectively improve the mechanical stability of the self-supporting electrode, thus ensuring stable operation over a long period of time [83,84]. (c) The morphology of in situ-grown catalysts can be easily modulated to effectively modify the hydrophilicity of the electrodes. Therefore, self-supported electrodes are more suitable for AWE at high current densities over long periods of time [85,86].

**Fig. 8.**
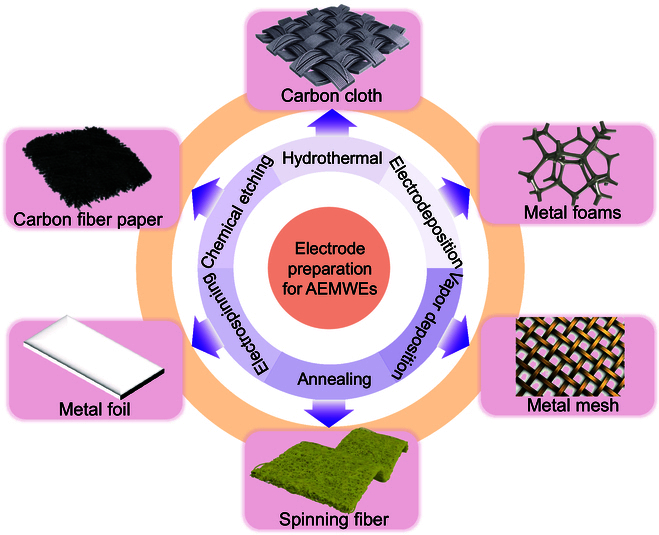
Manufacturing methods and substrates used for AEMWE systems’ self-supporting electrodes.

**Fig. 9.**
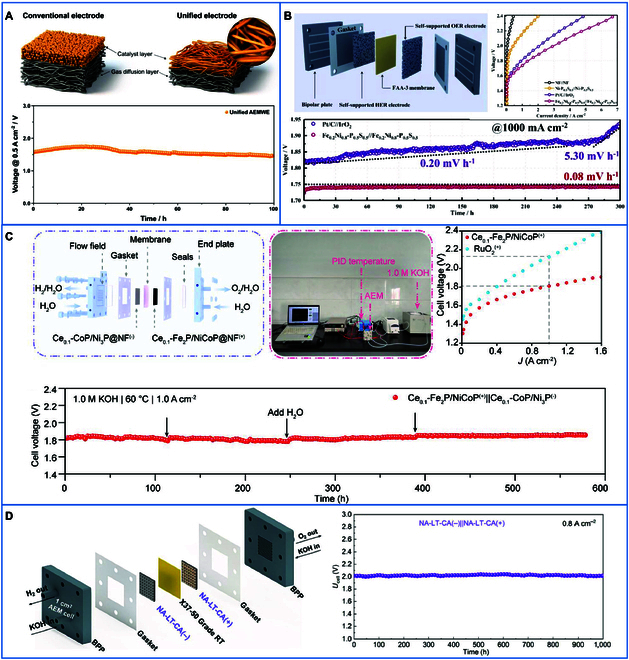
(A) Schematic diagram of the modified NiFeOOH electrode and durability test of AEMWE operation [98]. (B) Schematic diagram of AEMWE of Fe_0.2_Ni_0.8_-P_0.5_S_0.5_, polarization curve, and stability curve [99]. (C) Schematic diagram of the AEMWE electrolyzer of Ce_0.1_-Fe_2_P/NiCoP@NF, photograph of the AEMWE platform, polarization curve, and chronopotentiometry curve [36]. (D) AEMWE electrolyzer schematic diagram and durability test of NA-LT-Ca [10].

Self-supporting electrodes typically refer to an electrode material with a self-supporting structure that maintains its stability during application. As shown in Fig. 8, commonly used self-supporting substrates include carbon cloth [87–89], carbon fiber paper [90,91], metal foil [92,93], metal mesh (Ni/stainless steel mesh) [94,95], and metal foams (Ni/Fe foams) [3,96,97], among others. Specifically, the metal foil exhibits a one-dimensional (1D) structure, the metal mesh displays a 2-dimensional (2D) structure, and the carbon cloth, carbon paper, and metal foam manifest a 3-dimensional (3D) structure. The 3D structure enables the catalyst to have countless micropores and a large surface area. These features facilitate the generation of catalysts and the exposure of active sites, thus improving the catalytic efficiency. In addition, the 3D structure provides more space for the transport of reactants and diffusion of products. For instance, Park et al. [98] developed a 3D integrated electrode by directly electrodepositing NiFeOOH onto the GDL, enabling an AEMWE to operate stably for 100 h at 500 mA/cm^2^ and 24 h at 3,000 mA/cm^2^ (Fig. 9A). Similarly, Wan et al. [99] fabricated a Fe_0.2_Ni_0.8_-P_0.5_S_0.5_ quaternary catalyst via electrodeposition on Ni foam, achieving a current density of 2.5 A/cm^2^ at 2.0 V and stable operation for 300 h at 1,000 mA/cm^2^ in a MEA (Fig. 9B). Zhang et al. [36] demonstrated exceptional durability using a Ce_0.1_-Fe_2_P/NiCoP@NF anode, where the AEMWE operated for 579 h at 1.0 A/cm^2^ in 1.0 M KOH (60 °C) without significant degradation (Fig. 9C). Jiang et al. [10] further advanced this field by depositing NiAl alloy onto perforated Ni plates via atmospheric plasma spraying. This was followed by laser texturing to create a robust nickel-based electrode, which sustained 1,000 h of continuous operation at 0.8 A/cm^2^ in 1 M KOH at room temperature (Fig. 9D). Together, these studies underscore the critical role of self-supporting electrodes in attaining high current densities, long-term stability, and scalability for industrial AEMWE systems. The studies also underscore the necessity of further optimizing electrode architectures and interfacial engineering to address issues related to mass transport and mechanical durability.

To sum up, in order to obtain catalysts with higher catalytic activity, researchers usually adopt various methods to prepare them. Combining various preparation methods can benefit element doping, vacancy introduction, and lattice structure adjustment. This combination can also improve the catalyst’s physical properties, such as particle size, specific surface area, and pore volume, thus further optimizing the catalytic performance of the catalyst. In addition, various preparation methods are beneficial to the synthesis of multifunctional catalysts. By combining catalysts with different functions, multiple catalytic processes can be carried out simultaneously in a single reaction system. This broadens the application range of catalysts and improves their efficiency. This coupling and catalytic interaction requires precise design and control to ensure that each catalytic process can coordinate with each other and achieve the best catalytic effect.

### Ordered membrane electrodes

Ordered membrane electrodes (nanoscale imprinting, integrated membrane electrodes, and 3D interlocked interfaces) represent high-performance design paradigms targeting gap-free CLs/AEM contact and ordered transport channels to meet industrial current densities (>1 A/cm^2^). The conventional coating method for CL preparation involves blending catalysts with anion exchange ionomers (OH^−^ conductors) to form multiphase transport channels. However, the disordered distribution of OH^−^, electrons, gas, and water within these channels leads to significant electrochemical polarization and concentration polarization, which severely limits the high-current-density performance of MEAs. Although self-supporting electrodes eliminate the need for binders and enable the ordered growth of catalysts on GDL substrates, the lack of intimate contact between CLs and AEMs introduces interfacial resistance. This impedes the efficient transport of electrons and OH^−^ ions at the CLs/AEM interface. Achieving gapless bonding between CLs and AEM is critical for enabling ordered and rapid transport of electrons, OH^−^ ions, and water molecules, thereby enhancing MEA catalytic efficiency. However, the deposition of in situ CLs on polymer membranes presents challenges due to the nonconductive nature, solvent sensitivity, and limited thermal stability of AEM materials. These characteristics complicate direct catalyst anchoring and interfacial adhesion [73,100]. To address existing limitations, 3 advanced strategies for constructing gap-free ordered MEAs have been developed (Table 9): (a) nanoscale imprinting optimizes transport channels with anodic aluminum oxide (AAO) templates, achieving a 4.2 times higher active area, but is restricted to lab scale due to template fragility; (b) integrated membrane electrodes enable seamless CL-AEM interfaces via solvothermal synthesis, delivering 1,000 h stability at 1 A/cm^2^, yet struggle to scale beyond 100 cm^2^; and (c) ultrasonic-sprayed 3D interlocked interfaces eliminate delamination risks through binder-free designs, enabling > 1,800 h durability and roll-to-roll scalability for industrial production. While nanoscale imprinting aids material optimization and integrated electrodes excel in localized performance, the 3D architecture strikes the optimal balance between durability (low voltage decay) and scalability, positioning it as the most viable path toward commercializing high-performance AEMWE systems.

**Table 9.** Comparative analysis of advanced ordered MEA fabrication strategies

Strategy	Advantages	Challenges	Potential improvements
Nanoscale imprinting and template-assisted fabrication	• Enables ordered ionomer arrays (e.g., Nafion) with 4.2 times higher active area than conventional MEA. • Reduces mass transport polarization by 13.9% via gradient structures.	• Limited mechanical strength of templates (e.g., anodic aluminum oxide [AAO]). • Complex alignment processes for large-area fabrication.	• Develop hybrid templates with enhanced durability. • Optimize nanoimprinting parameters for roll-to-roll production.
Integrated membrane electrodes	• Achieves seamless CLs/AEM interfaces with minimal interfacial resistance. • Supports 1,000-h stability at 1,000 mA/cm^2^.	• Restricted to specific catalyst–substrate combinations. • Limited scalability due to solvothermal synthesis constraints.	• Expand in situ growth techniques to diverse catalysts. • Integrate solvent-resistant ionomers for wider pH ranges.
3D interlocked interfaces	• Reduces interfacial contact resistance (ICR < 10 mΩ·cm^2^) via vertical alignment. • Eliminates hot-pressing steps, enabling > 1,800-h durability.	• High equipment costs for ultrasonic spraying. • Limited control over ionomer distribution in 3D pores.	• Standardize spraying parameters for uniform coating. • Explore hybrid 3D printing for multimaterial integration.

#### Nanoscale imprinting and template-assisted fabrication

**Fig. 10.**
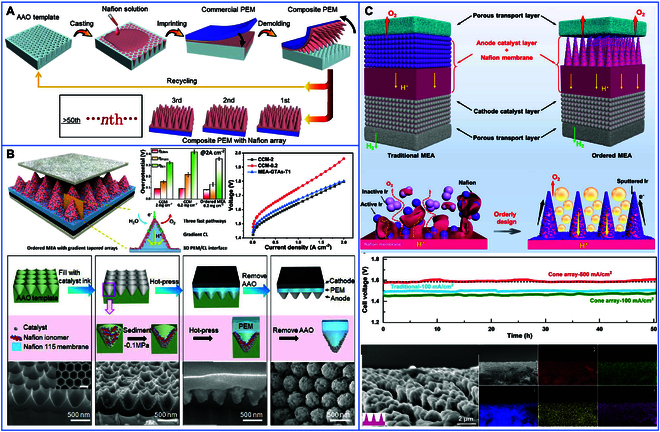
(A) Schematic of composite plasmonic exchange membrane with ordered Nafion arrays prepared cyclically by the nanoimprinting method [101]. (B) Polarization curves of the ordered MEA, SEM images of the preparation process, and the corresponding stages [102]. (C) Schematic diagram of the improvement from conventional MEA to ordered MEA, long-term stability test curves, and SEM images and corresponding EDS mapping images after durability operation [103].

These methods aim to optimize the contact tightness of CLs with AEM and the ordering of transport channels to improve the overall performance of the membrane electrode. As demonstrated in Fig. 10A, Li et al. [101] developed an efficient (<1 min), nonpolluting, and low-consumption nanoimprinting method to prepare large-area composite membranes with ordered Nafion arrays by recycling porous AAO templates. In an electrolytic cell, the performance of the above composite membranes is 1.6-fold higher than that of the corresponding commercial membranes, with a reduction of one-tenth of the hydrogen crossover rate. Similarly, Dong et al. [102] prepared membrane electrodes with 3D membrane/CLs interfaces and gradient tapered arrays using a nanoimprinting method (Fig. 10B). Thanks to the maximized 3-phase interface, fast proton transport, and gradient design of the CLs, this ordered structure stands out. With a loading of 0.2 mg/cm^2^, it dramatically increases the electrochemically active area by a factor of 4.2 compared to the conventional MEA, which has a loading of 2 mg/cm^2^. It also reduced the mass transport and ohmic polarization overpotentials by 13.9% and 8.7%, respectively. This ensures superior catalytic performance and stability. On this basis, as shown in Fig. 10C, Tian et al. [103] designed ordered MEAs by using an AAO template and the magnetron sputtering method. Ionic membranes with a surface cone-like structure markedly improved the utilization efficiency of the catalysts and reduced the loading to 20.0 μg/cm^2^. The CLs/PEM interface constructed by magnetron sputtering not only maintains the structure of Nafion arrays but also effectively increases the CLs/PEM interface interactions, thus providing abundant proton transfer pathways. In addition, the ordered array structure is vertically aligned with the liquid/porous transport layer and membrane, which facilitates the release of oxygen vesicles in the electrochemical reaction. As a result, these ordered MEAs achieved 8.7-fold higher electrochemically active area compared to conventional MEAs loaded with 1.0 mg/cm^2^. This nanoblotting method can be extended to the preparation of ordered AEMs, providing a larger contact area between the catalyst and the membrane and more favorable transport channels. These findings provide strong support for the preparation of ordered MEA in water electrolysis. Nevertheless, despite the demonstrated potential of template-assisted nanoimprinting in enhancing catalyst utilization and interfacial synergy, this technique faces inherent challenges for large-scale implementation. The process relies heavily on the fabrication of high-precision templates and multiple transfer steps, which result in elevated production costs and prolonged cycle times. Furthermore, achieving uniform electrode–membrane interfaces over large areas (e.g., >100 cm^2^) remains technically demanding due to alignment inaccuracies and material deformation during sequential imprinting. Future efforts should focus on developing scalable nanoimprinting approaches, such as roll-to-roll imprinting or hybrid laser-assisted self-assembly techniques, to address these limitations and facilitate the translation of ordered MEA designs from lab-scale breakthroughs to industrial-scale manufacturing.

#### Integrated membrane electrodes

**Fig. 11.**
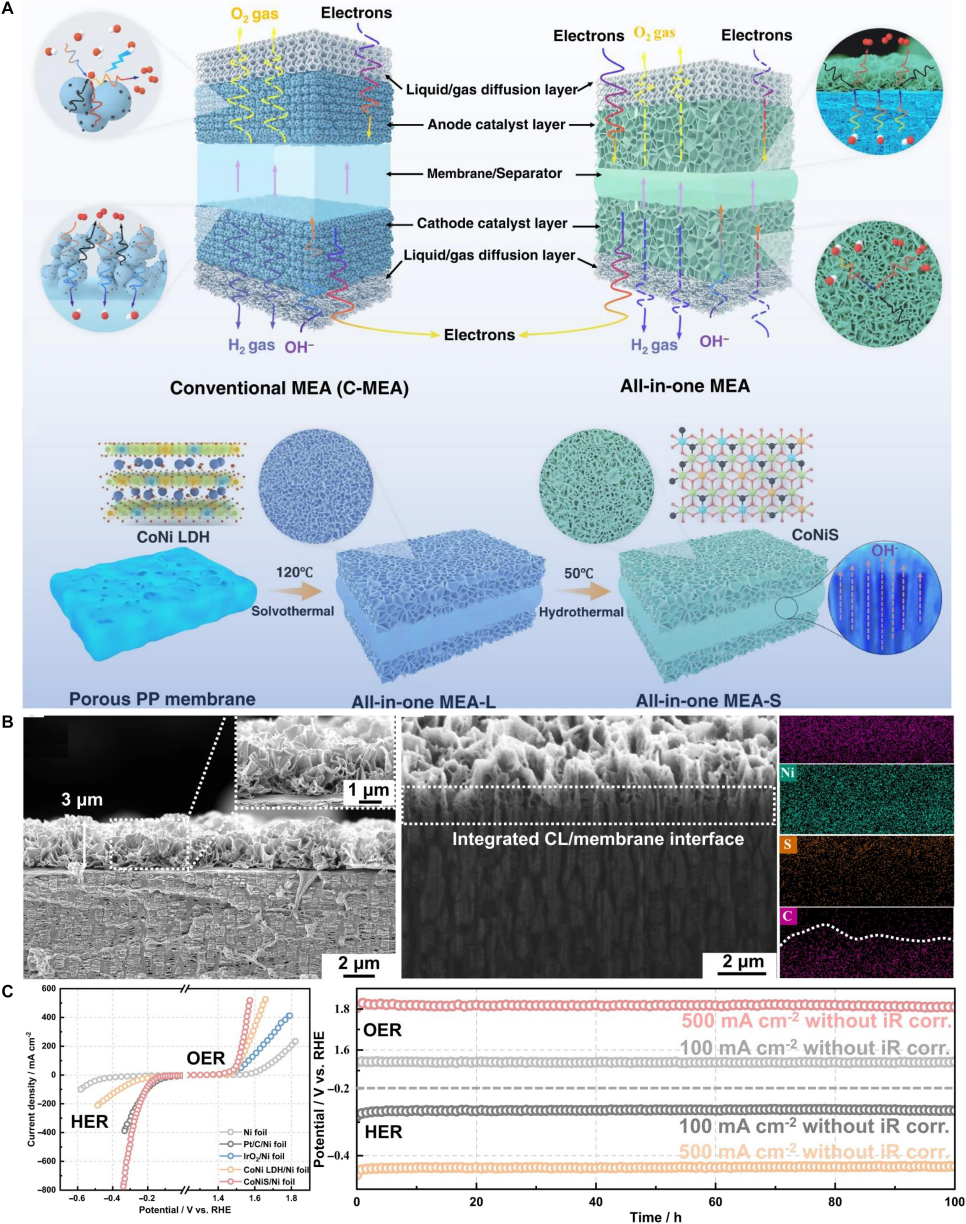
(A) Schematic of the preparation process of the integrated MEA. (B) Cross-sectional SEM image and corresponding EDX mapping of CLs in the integrated MEA. (C) Polarization curves and chronopotential curves of the electrodes [104].

The gradient membrane design optimizes membrane–CL contact tightness while expanding the catalyst–membrane interfacial area, thereby enhancing both efficiency and stability in water splitting. However, conventional CL thickness is constrained by binder limitations, as excessive thickness compromises catalyst–membrane contact quality and reaction kinetics. Moreover, the disordered microstructure of CLs reduces catalyst active site accessibility, further diminishing electrochemical performance. To address these challenges, Wan et al. [104] from Tsinghua University proposed an “integrated membrane electrode” concept, which synergizes intimate CLs/AEM interfacial contact with ordered catalyst architectures (Fig. 11A). By employing an in situ growth strategy of electrocatalysts within porous membranes, this design minimizes electron/gas/ion transport resistances and establishes ordered OH^−^ transfer channels. The solvothermal synthesis enables directional alignment of catalyst arrays on porous substrates, forming an integrated MEA with a high-surface-area ordered CL, low-curvature pore structures, and seamless CLs/AEM interfaces (Fig. 11B). This configuration achieved a current density of 1,000 mA/cm^2^ at 1.57 V (94% energy efficiency) in 30 wt% KOH and demonstrated >1,000-hour stability at 60 °C under 1,000 mA/cm^2^ (Fig. 11C), underscoring its potential for next-generation AWE systems. Despite these advancements, challenges persist in the scalable fabrication of integrated membrane electrodes. The solvothermal synthesis process, while effective for directional catalyst alignment, requires stringent reaction conditions that complicate large-area uniformity and industrial reproducibility. Moreover, the long-term mechanical integrity of the seamless CLs/AEM interfaces under dynamic operating stresses (e.g., membrane swelling) remains underexplored. Future studies should prioritize hybrid manufacturing strategies, such as combining in situ growth with roll-to-roll imprinting, to achieve cost-effective upscaling.

#### 3D interlocked interfaces

**Fig. 12.**
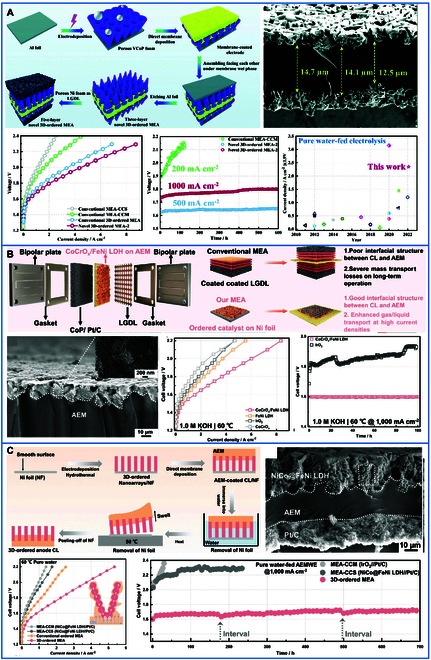
(A) Schematic diagram of stepwise synthesis of novel 3D ordered MEA by electrodeposition and direct film deposition methods; cross-sectional SEM images of membrane-coated VCoP-2 foams; electrochemical performance of VCoP-2 assembled into AEMWE [105]. (B) Schematic representation of MEA-designed AEMWE; cross-sectional SEM morphology; electrochemical performance of NiCo@FeNi LDH assembled into AEMWE [107]. (C) Schematic diagram of a novel expansion-assisted transfer method; cross-sectional SEM images of 3D ordered MEA based on NiCo@FeNi LDH porous foam as anodic CL; electrochemical performance of CoCrO*_x_*/FeNi LDH assembled into AEMWE [106].

In situ growth of catalysts on membranes is challenging because the catalyst deposition may block membrane pores, thereby impeding OH^−^ transport. To address this limitation, Wan et al. [105] developed an innovative approach using ultrasonic spraying to deposit membranes onto preformed CLs. This method not only enables precise control over membrane thickness (e.g., 5 to 10 μm with ± 0.5 μm accuracy) but also preserves membrane integrity by avoiding solvent-induced swelling. By adjusting the spraying direction relative to the CLs, they achieved perpendicular alignment between the electron transport channels and the AEM, markedly enhancing interfacial contact and reducing ICR. For instance, as illustrated in Fig. 12A, a highly ordered VCoP porous foam was synthesized on aluminum foil (AF) via electrodeposition. A polymer solution was then ultrasonically sprayed onto the VCoP/AF CLs surface to construct a 3D CLs/membrane layer (ML) interface. By pressing another VCoP/AF substrate before curing the membrane, a dual-sided 3D CL/ML structure was realized, maintaining the ordered porous nanostructure of CLs while reducing OH^−^ transport resistance. This process eliminated the need for hot-pressing, yet achieved robust adhesion between ML and CLs. The resulting AEMWE demonstrated exceptional performance, reaching a current density of 4,200 mA/cm^2^ at 2.0 V in 1 M KOH and sustaining stable operation for over 600 h at 1 A/cm^2^ under pure water supply. Building on this success, Wan et al. [106] further advanced their methodology by introducing a swelling-assisted transfer technique (Fig. 12B). They first fabricated a 3D ordered nanoarray of NiCo@FeNi LDH on smooth Ni foil through electrodeposition and hydrothermal synthesis. Quaternary ammonium poly(N-methyl-piperidine-co-p-terphenyl) (QAPPT) ionomer was then ultrasonically sprayed onto the porous nanoarray to form a uniform AEM layer. Subsequent swelling of the AEM in deionized water and heating at 50 °C facilitated the removal of the Ni foil, enabling seamless transfer of the nanoarray. A commercial Pt/C catalyst was sprayed onto the opposite side of the AEM to assemble a 3-layer MEA. This configuration achieved a current density of 3.61 A/cm^2^ at 2.0 V under pure water conditions and maintained stable operation for 700 h at 1.0 A/cm^2^ with minimal voltage decay (~1.7 V). Additionally, Wan et al. [107] applied ultrasonic spraying to deposit QAPPT polymer electrolyte onto CoCrO*_x_*/FeNi LDH nanosheet electrodes, creating a 3D interlocked CL/AEM interface (Fig. 12C). The aligned ionomer channels and vertical through-pores in the CL structure reduced overall cell resistance by 40% and enhanced liquid/gas mass transport. In 1 M KOH, the CoCrO*_x_*/FeNi LDH-based AEMWE achieved 3.91 A/cm^2^ at 1.8 V and sustained operation for 1,800 h at 1.0 A/cm^2^ in pure water, showcasing unparalleled durability. By integrating ultrasonic spraying with advanced structural engineering, Wan et al. have overcome critical challenges in AEMWE fabrication, such as OH^−^ transport bottlenecks and interfacial delamination. The ultrasonic spray deposition technique emerges as a highly promising strategy for scalable MEA fabrication, effectively bridging the gap between nanoscale interfacial engineering and industrial manufacturing requirements. By enabling precise thickness control, room-temperature processing, and compatibility with large-area substrates, this method circumvents traditional bottlenecks such as hot-pressing-induced membrane deformation and pore collapse. Crucially, the solvent-free nature and rapid deposition rates align with cost-effective mass production, while the ability to create 3D interlocked interfaces ensures performance retention during device scaling. Future research may focus on optimizing polymer–ionomer compatibility and scaling up these methods for industrial adoption.

## Summary and Outlook

AEMWE technology, with its unique advantages of combining the low cost of AWE and the high flexibility of PEMWE, is regarded as a core driver for the green hydrogen economy. This review compares and analyzes existing technological routes of hydrogen production by water electrolysis from comprehensive large-scale, long-life, and low-cost perspectives. It concludes that hydrogen production by water electrolysis with AEM technology has unique advantages. Since the MEA is the core of AEMWE, this review introduces the main factors affecting MEA performance, including the properties of CLs, AEM, and GDLs, as well as the construction of the 3-phase interface. Preparation techniques for MEA, including the preparation of powdered catalysts and self-supporting electrodes, are reviewed, highlighting the importance of ordered membrane electrode preparation. Although AEMWE has now achieved great success, many challenges remain to be urgently addressed.

1. Establishment of the standardized evaluation system

Current AEMWE research lacks unified performance evaluation criteria, leading to significant disparities in reported current densities (0.1 to 8 A/cm^2^), electrolyte concentrations (0.1 to 6 M KOH), and durability testing durations (100 to 12,000 h) across studies. These inconsistencies severely hinder technology benchmarking and industrial translation. For instance, branched poly(triphenyl piperidinium) AEM demonstrates high OH^−^ conductivity of 145 mS/cm at 80 °C, yet its testing conditions (1 M KOH, 80 °C) diverge from industrial-grade strong alkaline environments (6 M KOH). Future efforts must establish standardized testing frameworks based on industrial scenarios, such as mandating minimum stability testing requirements. Additionally, accelerated stress tests simulating real-world intermittent operation—including dynamic load cycling and thermal shock protocols—should be developed to bridge the gap between laboratory research and practical electrolyzer deployment.

2. Multiscale catalytic mechanism analysis and material innovation

The performance limitations of AEMWE are attributable to 2 primary factors. Firstly, there is a lack of clarity regarding the reaction kinetics at the catalyst–membrane interface and the material degradation mechanisms. Secondly, there is an inherent trade-off between the chemical stability and ion-conduction efficiency of AEMs themselves.

In the field of catalytic materials, nonprecious metal catalysts, such as NiFe oxyhydroxides, demonstrate high activity in laboratory settings. However, the oxidation–corrosion pathways of these catalysts under industrial current densities (>2 A/cm^2^) remain poorly defined. This leads to a progressive loss of active sites and interfacial passivation during long-term operation. For AEM, traditional quaternary ammonium groups are susceptible to Hofmann elimination in harsh alkaline and high-temperature environments (e.g., 80 °C, 6 M KOH), resulting in over 50% degradation in OH^−^ conductivity after 1,000 h of operation. While high ion exchange capacity membranes like HTMA-DAPP achieve elevated conductivity, they suffer from excessive swelling (swelling ratio > 30%) and microcrack propagation, which amplify hydrogen crossover risks. In order to address the aforementioned challenges, it is necessary to integrate multiscale modeling (e.g., density functional theory for cation leaching dynamics) with high-throughput experimentation (e.g., automated electrochemical workstations) to develop AEM materials. Such advancements would unravel molecular-level mechanisms behind material-interface synergy failures and pave the way for durable, high-performance components.

3. Industrial-scale fabrication of ordered membrane electrodes

The utilization of ordered membrane electrodes, engineered with vertical ion channels and 3D interlocked architectures, has been demonstrated to effectuate a reduction in mass transport resistance. However, the large-scale production of these electrodes is encumbered by challenges related to process complexity and cost. Current lab-scale methods, such as template-assisted electrodeposition, enable precise fabrication of highly active ordered structures but are equipment-intensive and low-yield, falling short of industrial throughput requirements. Industry efforts are now prioritizing simplified and intelligent manufacturing strategies. These include roll-to-roll coating technology enhanced by AI algorithms to optimize coating uniformity (>95%) and reduce noble metal ink consumption. However, critical barriers persist, such as reconciling high-temperature processing with flexible production workflows and mitigating ionomer delamination under humid–heat conditions. Achieving gigawatt-scale deployment of AEMWE electrolyzers and reducing green hydrogen costs below $1.5/kg will require synergistic innovation across materials, processes, and equipment. This will entail transitioning ordered membrane electrode fabrication from micron-scale precision to kilometer-scale productivity while maintaining performance integrity under industrial operating regimes.

In summary, the transition of AEMWE technology from laboratory breakthroughs to industrial applications necessitates the establishment of a standardized evaluation system encompassing materials, components, and systems. It also involves the analysis of the interface reaction mechanism under multiphysics coupling and the development of compatible intelligent preparation technology for flexible production. In addition, it is necessary to promote cross-field cooperation, accelerate the layout of global hydrogen energy infrastructure, and ultimately achieve the “dual carbon” goal and energy structure transformation.
